# Early Mortality in Patients With Muscle-Invasive Bladder Cancer Undergoing Cystectomy in the United States

**DOI:** 10.1093/jncics/pky075

**Published:** 2019-01-28

**Authors:** Kathryn E Marqueen, Nikhil Waingankar, John P Sfakianos, Reza Mehrazin, Scot A Niglio, François Audenet, Rachel Jia, Madhu Mazumdar, Bart S Ferket, Matthew D Galsky

## Abstract

**Background:**

Although radical cystectomy (RC) is a standard treatment for muscle-invasive bladder cancer (MIBC), for many patients the risks versus benefits of RC may favor other approaches. We sought to define the landscape of early postcystectomy mortality in the United States and identify patients at high risk using pretreatment variables.

**Methods:**

We identified patients with MIBC (cT2-T4aN0M0) who underwent RC without perioperative chemotherapy within the National Cancer Database (2003–2012). Using multistate multivariable modeling, we calculated time spent in three health states: hospitalized, discharged, and death more than 90 days postcystectomy. Cross-validation was performed by geographic region. Time spent in each state was weighted by utility to determine 90-day quality-adjusted life days (QALDs).

**Results:**

Among 7922 patients, 90-day mortality was 7.6% (8.0% for lower and 6.7% for higher volume hospitals). Increasing age, clinical T stage, Charlson Comorbidity Index, and lower volume were associated with higher 90-day mortality and were included in the model. Cross-validation revealed appropriate performance (C-statistics of 0.53–0.74; calibration slopes of 0.50–1.67). The model predicted 25% of patients had a 90-day mortality risk higher than 10%, and observed 90-day mortality in this group was 14.0% (95% CI = 12.5% to 15.6%). Mean quality-adjusted life days (QALDs) was 63 (range = 44–68).

**Conclusions:**

RC is associated with relatively high early mortality risk. Pretreatment variables may identify patients at particularly high risk, which may inform clinical trial design, facilitate shared decision making, and enhance quality improvement initiatives.

Radical cystectomy is a “gold standard” curative-intent treatment for muscle-invasive bladder cancer (MIBC) (1). However, radical cystectomy is a major operation, with life-altering implications due to the need for urinary diversion, and is associated with potentially considerable morbidity and mortality (2). Given that the majority of all bladder cancers are diagnosed in patients at least 65 years old (3), who often have smoking and/or age-related comorbidities, many patients are suboptimal candidates for radical cystectomy (4).

Delineating the landscape of early mortality postcystectomy is important for two key practical reasons.

1. Radiation therapy, with or without concurrent chemotherapy, is another potentially curative treatment option for MIBC (5). Prospective randomized trials comparing cystectomy and chemoradiation have proven unfeasible (6), and there are currently no standard approaches to identify patients who are more suitable for one treatment modality versus the other. Indeed, chemoradiation in the United States is considered the preferred default approach, as per national treatment guidelines, for “noncystectomy candidates” (7) or “those with significant comorbidities for whom radical cystectomy is not a treatment option.” (8) However, no uniform definition of the “noncystectomy candidate” exists that may inform clinical care or form the basis for eligibility criteria for future clinical trials.

2. Enhanced Recovery After Surgery (ERAS) strategies have emerged, which may improve perioperative outcomes in patients undergoing cystectomy (9). However, such strategies have predominantly been limited to higher volume centers and require dedicated teams and resources that may be best deployed and/or refined for “at risk” patients. Identifying patients at particularly high risk for early postcystectomy mortality, therefore, could have important implications regarding shared decision making, clinical trial design, resource utilization, quality improvement initiatives, and policy making.

Herein, we utilized a large observational dataset of patients with MIBC undergoing cystectomy in the United States to define the landscape of early postcystectomy mortality and to develop a state-transition model to identify patients at very high risk using routinely available precystectomy variables.

## Methods

### Data

This study analyzed data derived from the National Cancer Database (NCDB). The NCDB was created jointly by the Commission on Cancer (CoC) of the American College of Surgeons and the American Cancer Society as a registry comprising data from more than 1500 hospitals with CoC-accredited cancer programs in the United States and includes approximately 70% of all newly diagnosed cases of cancer (10). This study was deemed exempt by the Icahn School of Medicine at Mount Sinai Institutional Review Board.

### Selection of Study Population

**Figure 1.**
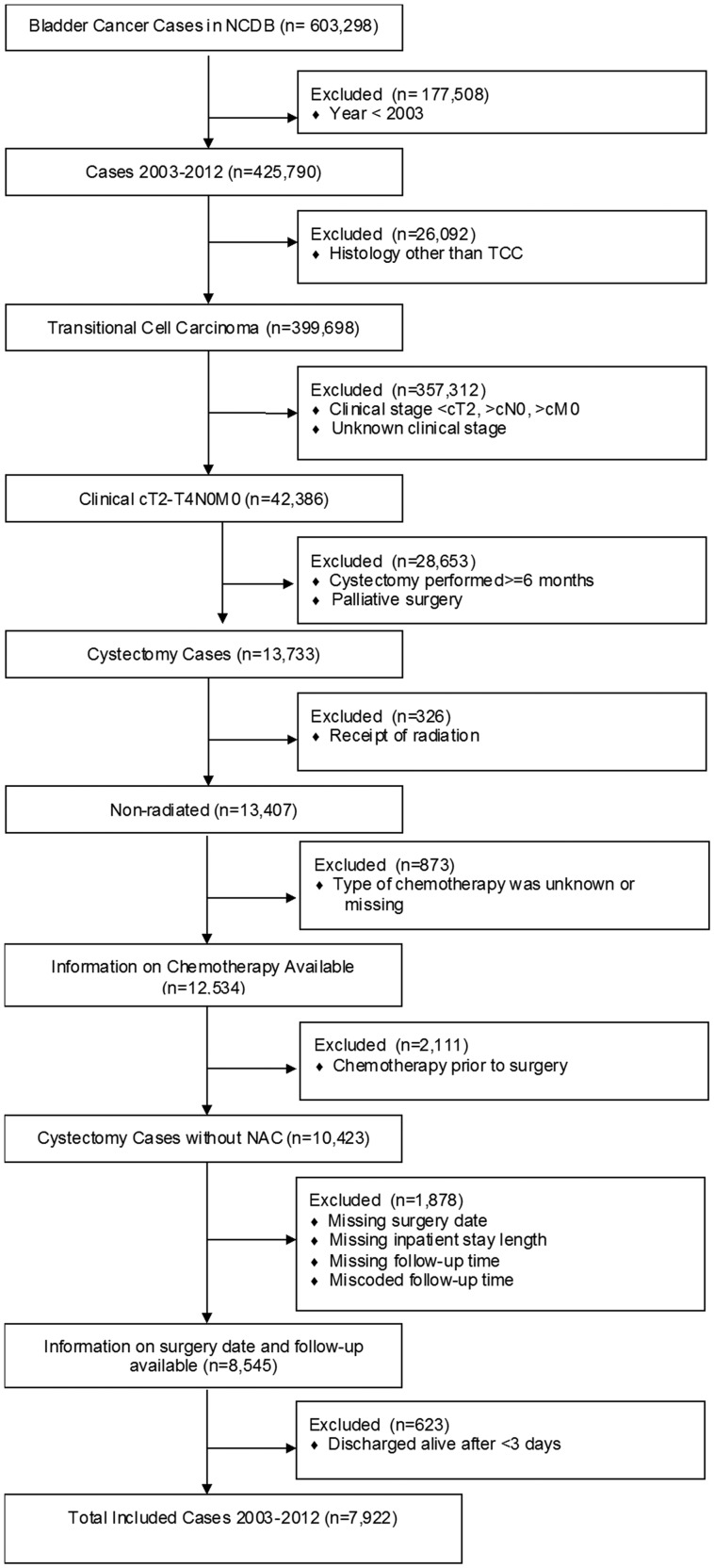
Selection of study population with reasons for patient exclusions. NCDB = National Cancer Database; TCC = transitional cell carcinoma.

The NCDB was queried to identify all patients who were diagnosed with American Joint Committee on Cancer (AJCC) TNM stage cT2–T4aN0M0 urothelial cancer of the bladder between 2003 and 2012 and who underwent cystectomy as the primary curative treatment within 6 months of clinical diagnosis. Patients were excluded based on receipt of radiation or neoadjuvant chemotherapy, palliative intent of surgery, and wrong or missing follow-up information, including time to discharge, resulting in a final study population of 7922 patients (Figure 1 ).

### Predictor and Outcome Variables

Patient-, tumor-, and facility-level covariates were considered for inclusion in the prediction model based on previous literature as well as availability in the NCDB and clinical practice. Patient-level candidate predictors included age, sex, and Charlson/Deyo Comorbidity Index (CCI) defined by the NCDB into 0, 1, and at least 2. Clinical T (cT) stage was included as a tumor-level variable. Facility-level candidate predictors included cystectomy volume, which was categorized according to approximate tertiles of the number of cystectomies (<5, 5–14, ≥15) performed by each reporting facility in the year preceding the patient’s year of diagnosis. To further evaluate the impact of cystectomy volume on outcomes, we additionally fitted prediction models including more granular volume categories. Outcomes included death within 90 days of cystectomy and hospital discharge. Death within 90 days of cystectomy was determined using the date of surgery and date of last contact or death, with patient vital status distinguishing contact and death for the latter date. Time to discharge was defined by the NCDB as the number of days between the date of cystectomy and hospital discharge.

### Missing Data

Missing predictor values were imputed by multiple imputation (MI = 20) with a flexible additive model including all predictors, status variables for discharge and death, and the Nelson–Aalen estimators of the cumulative hazard for discharge and death (11). Rubin’s rules were used to summarize the effect estimates and variances across multiple imputed datasets.

### State-Transition Modeling of Early Postcystectomy Mortality

**Figure 2.**
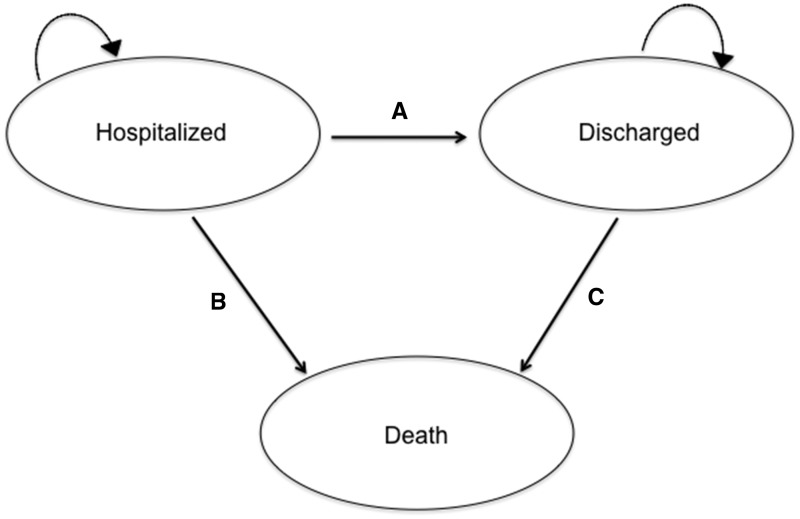
State-transition diagram of the multistate model. Each bubble represents a possible state, with arrows representing possible transitions (A = discharge; B = inpatient death; C = postdischarge death) with each 1-day cycle. All patients started in the hospitalized state, with death being a terminal state.

Because the NCDB does not define cause of death, in an attempt to enrich for cystectomy-related deaths versus cancer-related deaths, we selected a 90-day time horizon from cystectomy based on existing literature (12) and the observed distribution of the mortality hazard rate following cystectomy in our study population (Supplementary Figure 1, available online). We developed a state-transition model calculating time from radical cystectomy until 90 days or death, taking into account timing of discharge (13). As illustrated in Figure 2 , the model consisted of three states: hospitalized, discharged, and death, with all patients beginning in the hospitalized state and with death being a terminal state. The model had a 1-day cycle length, assuming events occur halfway through each cycle (14). Transition probabilities used in the model were calculated from state-transition hazard rates using time since cystectomy as the timescale. The state-transition model was leveraged to estimate not only early postcystectomy mortality but also 90-day quality-adjusted life days (QALDs) (15). This outcome was derived from time from cystectomy to discharge and death due to any cause within 90 days following cystectomy. Time spent in each of the model states (hospitalized, discharged, death) was weighted by multiplying duration in each state by utility scores for similar health states derived from the literature (0.67 for hospitalized, 0.77 for discharged, 0 for death) (16).

The state-transition equations were individualized using a set of Cox regressions predicting covariate effects on the hazard rate for each state transition (discharge, inpatient death, postdischarge death) with time since cystectomy as the timescale while censoring subjects who were lost to follow-up or at 90 days. Final Cox regression models were selected from full models including all candidate predictors in a backward stepwise manner using the Akaike’s Information Criterion as selection criterion.

For validation of the regression equations, we performed an internal–external cross-validation procedure in which the dataset was separated by facility location (N = 6 geographical regions). Each regression equation was refitted in data defined by five geographical regions, leaving one region out. Performance of refitted regression equations was then assessed in the region left out with the C-statistic and the calibration slope (1 = optimal) (17,18). Because there were six geographical regions in total, this procedure was performed six times with validation in each region.

All analyses were performed using R software version 3.3.2 (R Foundation for Statistical Computing, http://www.r-project.org/).

## Results

### Study Cohort Characteristics

The baseline characteristics of the 7922 patients eligible for inclusion are detailed in Table 1

**Table 1.** Study population (N = 7922) baseline characteristics. Data are presented as mean ± standard deviation or median (interquartile range) for continuous variables and as frequencies (%) for categorical data

Variable	cT2 (N = 6468)	cT3 (N = 981)	cT4 (N = 473)
Age, mean±SD, y	69.0 ± 10.4	70.6 ± 10.2	69.7 ± 10.3
Male, no. (%)	4958 (77)	682 (70)	403 (85)
Race, no. (%)			
White	5839 (90)	889 (91)	417 (88)
Black	320 (5)	41 (4)	26 (5)
Hispanic	103 (2)	25 (3)	8 (2)
Other	148 (2)	16 (2)	17 (4)
Missing	58 (1)	10 (1)	5 (1)
Insurance status, no. (%)			
Medicaid	204 (3)	37 (4)	20 (4)
Medicare	4022 (62)	625 (64)	311 (66)
Not insured	174 (3)	23 (2)	11 (2)
Other Government	42 (0.6)	6 (0.4)	2 (0.4)
Private	1962 (30)	275 (28)	124 (26)
Missing	64 (1)	15 (2)	5 (1)
Median income, no. (%)			
<$30 000	736 (11)	99 (10)	54 (11)
$30 000–$34 999	1207 (19)	185 (19)	99 (21)
$35 000–$45 999	1844 (29)	267 (27)	140 (30)
≥$46 000	2394 (37)	378 (39)	156 (33)
Missing	287 (4)	52 (5)	24 (5)
Without high school education, no. (%)*			
>29%	866 (13)	122 (12)	71 (15)
20–28.9%	1467 (23)	230 (23)	114 (24)
14–19.9%	1595 (25)	238 (24)	129 (27)
<14%	2253 (35)	338 (34)	135 (29)
Missing	287 (4)	52 (5)	24 (5)
Charlson Comorbidity Index, no. (%)			
0	4431 (69)	666 (68)	332 (70)
1	1567 (24)	249 (25)	101 (21)
≥2	517 (7)	66 (7)	40 (8)
Year of diagnosis, no. (%)			
2003	474 (7)	74 (8)	45 (10)
2004	442 (7)	74 (8)	29 (6)
2005	524 (8)	81 (8)	32 (7)
2006	562 (9)	96 (10)	37 (8)
2007	672 (10)	95 (10)	42 (9)
2008	1012 (16)	180 (18)	68 (14)
2009	1147 (18)	182 (19)	71 (15)
2010	855 (13)	109 (11)	80 (17)
2011	780 (12)	90 (9)	69 (15)
Facility type, no. (%)			
Community Cancer Program	389 (6)	49 (5)	37 (8)
Comprehensive Community Cancer Program	2778 (43)	464 (47)	223 (47)
Academic/Research Program	3283 (51)	465 (47)	213 (45)
Other	18 (0.3)	3 (0.3)	0 (0)
Facility location, no. (%)			
New England/Middle Atlantic	1300 (20)	227 (23)	108 (23)
South Atlantic	1062 (16)	187 (19)	99 (21)
East North Central	1287 (20)	202 (21)	94 (20)
East South Central/West South Central	910 (14)	121 (12)	67 (14)
West North Central	733 (11)	92 (9)	43 (9)
Mountain/Pacific	1176 (18)	152 (15)	62 (13)
Facility setting, no. (%)			
Rural	171 (3)	23 (2)	10 (2)
Urban	1200 (19)	164 (17)	102 (22)
Metro	4822 (75)	751 (77)	331 (70)
Missing	275 (4)	43 (4)	30 (6)
Annual cystectomy volume, no. (%)	10 (4–24)	8 (4–17)	8 (4–17)
Low (<5)	1362 (21)	274 (28)	122 (26)
Intermediate (5–14)	2185 (34)	360 (37)	181 (38)
High (≥15)	2387 (37)	272 (28)	139 (29)
Missing	534 (8)	75 (7)	31 (7)
Time to discharge, d	8 (7–12)	8 (7–12)	8 (7–13)

*Percentage of persons with less than a high school education within the patient’s census tract of residence. cT = clinical T stage.

**Figure 3.**
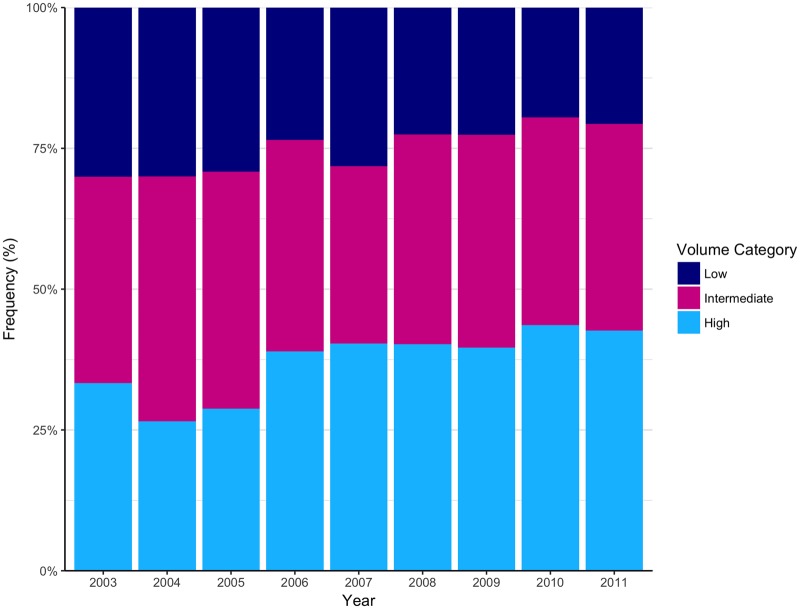
Proportion of cystectomies by volume category over time. The graph depicts the proportion of patients who underwent cystectomy in each of the volume categories separated by year of diagnosis. Volume categories were determined by the number of cystectomies performed by the institution in the year preceding the patient’s year of diagnosis.

. The median age of the study population was 69 years (interquartile range: 62–77), and median length of hospitalization was 8 days (interquartile range: 7–12). Within the cohort, 1758 (22.2%) patients underwent cystectomy at a lower volume hospital (<5 cases per year), 2726 (34.4%) at an intermediate volume hospital (5–14 cases per year), and 2798 (35.3%) at a higher volume hospital (≥15 cases per year). The proportion of cystectomies by volume category over time is shown in Figure 3 , demonstrating that even in the most recent year of the analysis, approximately 20% of patients underwent cystectomy at lower volume hospitals.

### Early Postcystectomy Mortality

The observed 90-day mortality rate in the entire study cohort was 600 of 7922 (7.6%). When stratified by annual cystectomy volume, the observed 90-day mortality rate was 140 of 1758 (8.0%), 220 of 2726 (8.1%), and 187 of 2798 (6.7%) at lower, intermediate, and higher volume hospitals, respectively. The observed 90-day mortality rate was 44 of 475 (9.3%) in community cancer centers, 291 out of 3465 (8.4%) in comprehensive community cancer centers, and 265 of 3961 (6.7%) in academic centers. The 90-day mortality by year of cystectomy is demonstrated in Supplementary Figure 2 (available online) and was relatively stable during the study period.

### State-Transition Model

The adjusted hazard ratios (HRs) for each state transition are shown in Table 2

**Table 2.** Multivariable adjusted hazard ratios (HRs) for discharge or death within 90 days of cystectomy

	Multivariable HR (95% CI)*		
Predictor	Discharge	Inpatient Death	Post-discharge Death
Age	0.991 (0.989 to 0.993)	1.044 (1.027 to 1.062)	1.060 (1.049 to 1.072)
Male	1.054 (1.000 to 1.111)	—	—
Charlson Comorbidity Index			
0	Referent	Referent	Referent
1	0.933 (0.884 to 0.984)	1.453 (1.054 to 2.002)	1.533 (1.241 to 1.893)
≥2	0.918 (0.841 to 1.003)	2.192 (1.389 to 3.459)	1.696 (1.229 to 2.340)
Clinical T stage			
cT2	Referent	—	Referent
cT3	0.954 (0.891 to 1.022)	—	1.507 (1.168 to 1.944)
cT4	0.922 (0.838 to 1.015)	—	1.892 (1.365 to 2.622)
Annual cystectomy volume			
<5	Referent	Referent	—
5–14	0.997 (0.919 to 1.040)	0.892 (0.621 to 1.282)	—
≥15	1.116 (1.051 to 1.185)	0.639 (0.425 to 0.960)	—

*When HRs are omitted, variables were not selected in the final model. CI = confidence interval.

with inclusion of a more granular covariate for cystectomy volume in Supplementary Table 1 (available online). Increasing age, female gender, lower cystectomy volume, and higher CCI were associated with a longer time to hospital discharge. Increasing age, higher CCI, and undergoing cystectomy at a lower volume hospital were associated with greater risk of inpatient mortality within 90 days of cystectomy. Increasing age, higher cT stage, and higher CCI were associated with a greater risk of postdischarge mortality within 90 days of cystectomy. The model showed generally transportable discriminative ability and calibration slopes across geographical regions for each of the three state transitions, with C-statistics of 0.53–0.74 and calibration slopes of 0.50–1.67 (Supplementary Table 2, available online).

**Figure 4.**
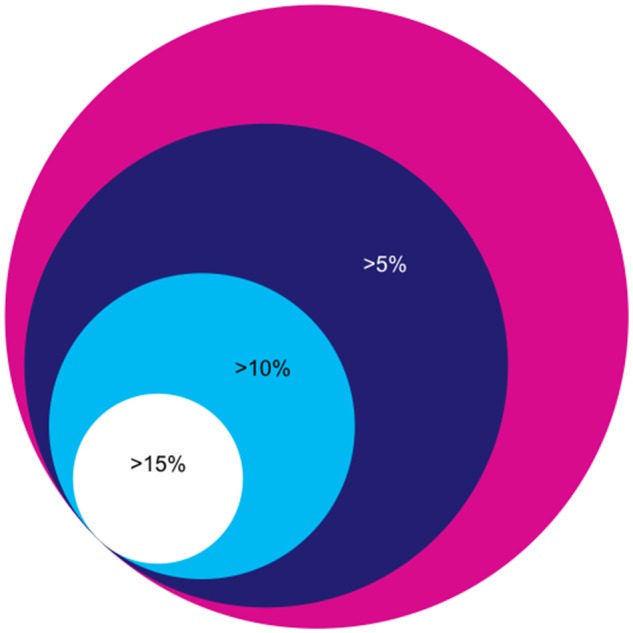
Proportional area diagram of predicted 90-day risk of mortality. The area of each circle is proportional to the number of patients in each labeled risk category (predicted 90-day mortality >5%, >10%, or >15%). The largest circle represents all patients in the study population.

The model predicted that 25% of patients in the cohort were at very high risk for early postcystectomy mortality (ie, 90-day mortality risk >10%; Figure 4 ), with observed 90-day mortality in this group of 14.0% (95% CI = 12.5 to 15.6). The predicted 90-day mortality risk distribution by hospital cystectomy volume category is shown in Supplementary Figure 3 (available online).

**Figure 5.**
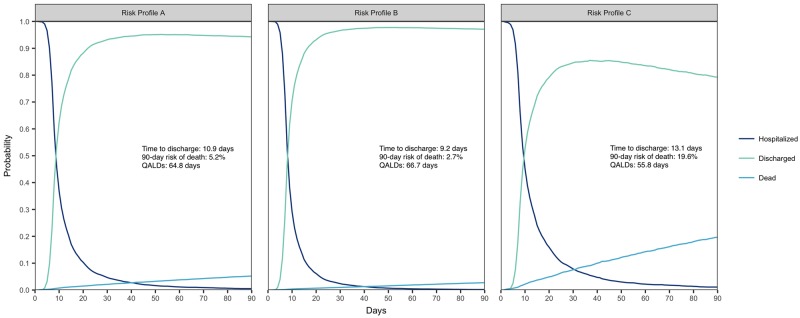
State-transition model-based predictions using individual patient risk profiles as examples: A) Patient with most common values: 69-year-old Caucasian male with clinical T (cT) stage 2 disease; Charlson/Deyo Comorbidity Index (CCI) = 0; cystectomy volume category = intermediate (5–14 cases per year). B) 60-year-old Caucasian male with cT2 disease; CCI = 0; cystectomy volume category = high (≥15 cases per year). C) 80-year-old Caucasian female with cT3 disease; CCI = 1; volume category = low (<5 cases per year). CCI = Charlson/Deyo Comorbidity Index; cT = clinical T stage; QALDs = quality-adjusted life days.

The mean predicted QALDs in the overall cohort was 63.4 and ranged from 44.0 to 68.2. Variations in predicted QALDs and risk of 90-day mortality according to baseline characteristics are demonstrated with three case examples in Figure 5. For example, patient A represents a 69-year-old man with the most common baseline characteristics in the cohort. A patient with this risk profile is predicted to have QALDs of 64.8 and 90-day risk of mortality of 5.2%. Patient B represents a 60-year-old man with cT2 disease and a CCI of 0, who underwent cystectomy at a higher cystectomy volume institution resulting in predicted QALDs of 66.7 and predicted 90-day risk of mortality of 2.7%. In contrast, patient C is an 80-year-old woman with a higher CCI of 1, undergoing cystectomy at a lower cystectomy volume hospital, whose predicted QALDs is 55.8 and predicted 90-day risk of mortality is 19.6%.

## Discussion

Muscle-invasive urothelial cancer of the bladder is a disease characterized by an aggressive biology with approximately 50% of patients developing lethal metastatic recurrence despite undergoing potentially curative surgery (19). At the same time, the disease most commonly occurs in elderly patients, who often have multiple comorbidities. Striking the appropriate balance between the risks and benefits of cystectomy is therefore a highly complex task in patients simultaneously facing competing risks of cancer-related, comorbidity-related, and treatment-related morbidity and mortality. Further complicating treatment decisions is the availability of an alternative treatment modality, radiation therapy, that has never been fully embraced in the United States at least in large part because of a lack of definitive comparative effectiveness data.

Here, we sought to define the landscape of early mortality and quality-adjusted survival in patients with MIBC undergoing cystectomy and identify patients at particularly high risk using a large observational cohort from a database capturing more than 70% of incident cases of cancer in the United States. Our analysis reveals several key findings. Radical cystectomy is associated with a relatively high 90-day mortality rate despite improvements in supportive care, surgical technology, and centralization of care. The observed 90-day mortality rate remained similar year by year during the time period encompassed in our study. Using a state-transition model, we identified that routinely available preoperative variables including increasing age, higher cT stage, and higher CCI, and undergoing cystectomy at a lower volume hospital were associated with a greater risk of mortality within 90 days of cystectomy. Strikingly, our model predicted that 25% of this large patient cohort, derived from data representing the continuum of practice settings in the United States, was at very high risk (>10%) of 90-day mortality and that the observed 90-day mortality in this very high-risk subgroup was 14%.

Our analysis is by no means an indictment of cystectomy, which remains, in the absence of comparative effectiveness data, a backbone of curative intent treatment for the majority of patients with MIBC. It is also not our goal to refute data that suggests cystectomy may be underutilized in select elderly patients (20). Rather, our results may help refine and risk-stratify the care of individual patients with MIBC in several practical ways. Treatment options for MIBC require trade-offs, which may potentially include higher risks of early mortality in exchange for potentially improved longer-term outcomes, and such trade-offs may be viewed differently by patients in the context of their individual circumstances. Currently, practice guidelines recommend chemoradiation for noncystectomy candidates with MIBC. However, no uniform definition of the noncystectomy candidate exists, which hampers standardization of practice and perhaps most importantly the development of reproducible eligibility criteria for prospective clinical trials seeking to optimize the care of this large patient population. Although admittedly somewhat arbitrary, a predicted postcystectomy 90-day mortality of more than 10% and/or QALDs less than 60 (eg, patient C in Figure 5) utilizing our model could be considered for such a definition. ERAS and prehabilitation strategies have recently been introduced into the perioperative management of patients undergoing cystectomy, which may further improve patient outcomes, including early mortality (21–24). Identification of patients at very high risk for early morbidity and mortality may facilitate resource allocation and auditing of such programs as well as quality improvement efforts. Nonetheless, ERAS programs have been mainly limited to higher volume cystectomy hospitals threatening to further widen the gap in outcomes compared with lower volume centers. Taken together, one could envision policy implications of our findings such that patients at very high risk for early postcystectomy mortality at lower volume facilities are either triaged to higher volume facilities or to treatment with radiation-based strategies optimized through prospective clinical trials.

There are several strengths associated with our study. We utilized a large observational dataset and included pretreatment variables that are routinely available. We employed a state-transition model that incorporates both overall survival and quality-adjusted survival over a relevant time horizon. We assessed our model’s performance, and adequate discrimination and calibration were demonstrated across different geographical regions.

There are potential weaknesses of our study. In particular, our cohort represents a time period prior to the routine use of ERAS protocols. Along with improvements in surgical technology (eg, robotic cystectomy), these protocols featuring holistic approaches to decrease perioperative morbidity and mortality have likely lowered the risk for early mortality after radical cystectomy compared with the time period studied. However, despite the centralization of cystectomy care, 51% of our cohort underwent cystectomy at community hospitals and 27% underwent cystectomy at lower volume hospitals. We did not have information regarding cause of death and therefore could not definitively distinguish between cancer and treatment-related deaths. However, we observed a bimodal hazard for death postcystectomy with an initial peak at day 12 followed by a nadir at approximately day 90, strongly suggestive of a period of increased early treatment-related death followed later by an increase in the hazard of presumably cancer-related death (Supplementary Figure 1, available online). Clinical stage was among the pretreatment variables associated with early mortality further complicating the ability to dissect treatment- and cancer-related deaths. However, prolonged anesthesia time and the morbidity associated with larger primary tumors may potentially explain this observation because cT stage was also associated with a longer length of hospital stay. Furthermore, because of the retrospective nature of this study, for a proportion of patients, risk factor status may have been misclassified, potentially biasing associations of the candidate predictors with the outcomes. However, our study included a large number of events relative to the number of predictive variables, increasing the precision and generalizability of our predictions. Missing data may also present a limitation to this study. However, similar results were obtained following multiple imputation of missing predictor values. Overall, there remains the need for further data collection, especially through multisite prospective registries.

In conclusion, we developed a multistate model to define early mortality and quality-adjusted survival in patients undergoing cystectomy using a large observational dataset. We demonstrated that a large subgroup of patients at very high risk for early postcystectomy mortality may be identified using pretreatment variables that are routinely available. Our model may have utility for the development of uniform criteria to define the noncystectomy candidate, to facilitate shared patient-physician decision making, to refine ERAS approaches, and to inform policy.

## Funding

This work was supported by the National Cancer Institute at the National Institutes of Health (P30 CA196521–01 to MDG, BSF, MM, RJ); the Biostatistics Shared Resource Facility, Icahn School of Medicine at Mount Sinai (MM, RJ, BSF); the American Heart Association (#16MCPRP31030016 to BSF); and the Alpha Omega Alpha Honor Medical Society (2017 Carolyn L. Kuckein Student Research Fellowship to KEM).

## Notes

Affiliations of authors: Division of Hematology/Oncology, Department of Medicine, Tisch Cancer Institute (KEM, SAN, MDG), Department of Urology (NW, JPS, RM, FA), and Department of Population Health Science and Policy, Institute for Healthcare Delivery Science (RJ, MM, BSF), Icahn School of Medicine at Mount Sinai, New York, NY

Conflicts of interest disclaimer: John P. Sfakianos: Consulting or advisory role: EMD Serono; Speakers’ bureau: Astellas Medivation. Matthew D. Galsky: Consulting or advisory role: BioMotiv, Janssen, Dendreon, Merck, GlaxoSmithKline, Lilly, Astellas Pharma, Genentech; Speakers’ bureau: Bristol-Myers Squibb; Travel, accomodations, expenses: BioMotiv, Merck, Dendreon, Astellas Pharm; Patents, royalties, other intellectual property: Methods and compositions for treated cancer and related methods, Mount Sinai School of Medicine, July 2012, application No. 20120322792; Stock and other ownership interests: Dual Therapeutics; Research funding: Janssen Oncology (Inst), Dendreon (Inst), Novartis (Inst), Bristol-Myers Squibb (Inst), Merck (Inst). All other authors have no relationships to disclose.

Disclaimer: The data used in the study are derived from a de-identified NCDB file. The American College of Surgeons and the Commission on Cancer have not verified and are not responsible for the analytic or statistical methodology employed or the conclusions drawn from these data by the investigator.

## Supplementary Material

Supplementary Data
