# Supplementary material for: Early Mortality in Patients With Muscle-Invasive Bladder Cancer Undergoing Cystectomy in the United States
Source: JNCI Cancer Spectr. 2019 Jan 28;2(4):pky075. doi: 10.1093/jncics/pky075 (PMC6349610; doi:10.1093/jncics/pky075)

**Supplement: Early mortality in patients with muscle-invasive bladder cancer undergoing cystectomy in the United States**

Supplemental Table 1:

|  | **Multivariable HR (95% CI)*** | | |
| --- | --- | --- | --- |
| Predictor | Discharge | Inpatient Death | Post-discharge Death |
| Age | 0.991 (0.986-0.993) | 1.045 (1.028-1.063) | 1.060 (1.049-1.072) |
| Male | 1.058 (1.004-1.116) | - | - |
| Charlson Comorbidity Index |  |  |  |
| 0 | ref | ref | ref |
| 1 | 0.931 (0.883-0.982) | 1.417 (1.026-1.957) | 1.533 (1.241-1.893) |
| 2+ | 0.915 (0.838-1.000) | 2.198 (1.393-3.468) | 1.696 (1.229-2.340) |
| Clinical T stage |  |  |  |
| cT2 | ref | - | ref |
| cT3 | 0.954 (0.891-1.022) | - | 1.507 (1.168-1.944) |
| cT4 | 0.922 (0.838-1.015 ) | - | 1.892 (1.365-2.622) |
| Annual cystectomy volume |  |  |  |
| <5 | ref | ref | - |
| 5-9 | 0.962 (0.903-1.025) | 0.830 (0.567-1.215) | - |
| 10-14 | 0.960 (0.890-1.035) | 0.941 (0.602-1.469) | - |
| 15-24 | 1.019 (0.945-1.099) | 0.468 (0.256-0.853) | - |
| 25+ | 1.204 (1.088-1.237) | 0.686 (0.445-1.057) | - |

Supplemental Table 2: Cross-validity of Cox Regression Models

| Facility location | **Discharge** | | **Inpatient Death** | | **Post-discharge Death** | |
| --- | --- | --- | --- | --- | --- | --- |
|  | **C-statistic** | **Calibration Slope** | **C-statistic** | **Calibration Slope** | **C-statistic** | **Calibration Slope** |
| Northeast/Middle Atlantic  (N=1,790) | 0.56 | 1.03 | 0.67 | 1.02 | 0.68 | 0.97 |
| South Atlantic  (N=1,474) | 0.55 | 0.57 | 0.68 | 1.25 | 0.68 | 1.05 |
| East North Central  (N=1,710) | 0.57 | 1.25 | 0.63 | 0.91 | 0.68 | 0.93 |
| East South Central/West South Central  (N=1,200) | 0.57 | 1.67 | 0.63 | 0.92 | 0.64 | 0.70 |
| West North Central  (N=938) | 0.56 | 0.56 | 0.50 | 0.50 | 0.74 | 1.46 |
| Mountain/Pacific  (N=1,522) | 0.53 | 0.51 | 0.64 | 0.75 | 0.65 | 0.83 |

Supplemental Figure 1: Mortality hazard rate following cystectomy. The shaded region represents the 95% confidence interval.


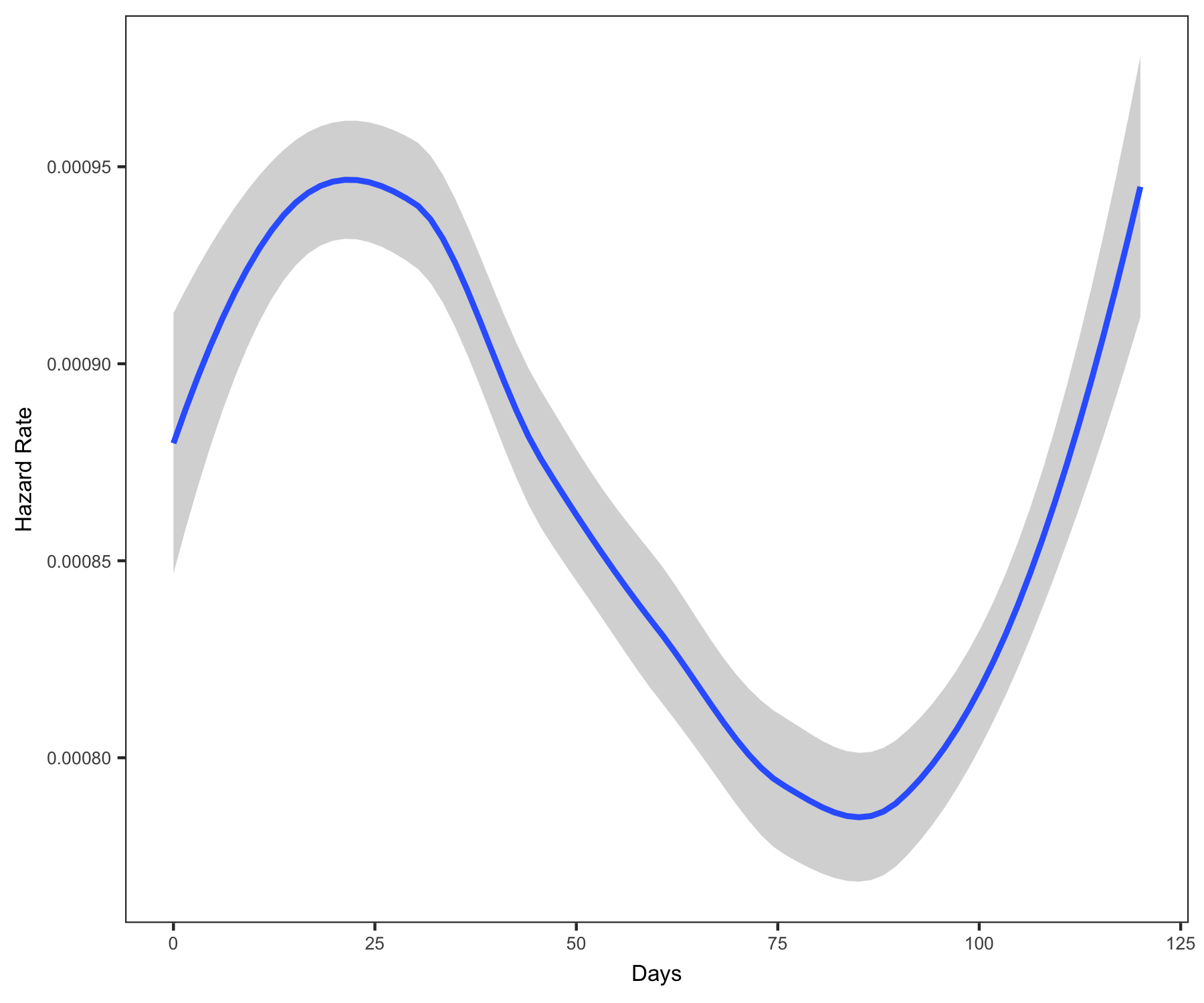


Supplemental Figure 2: Observed 90-day mortality (%) by year of diagnosis.


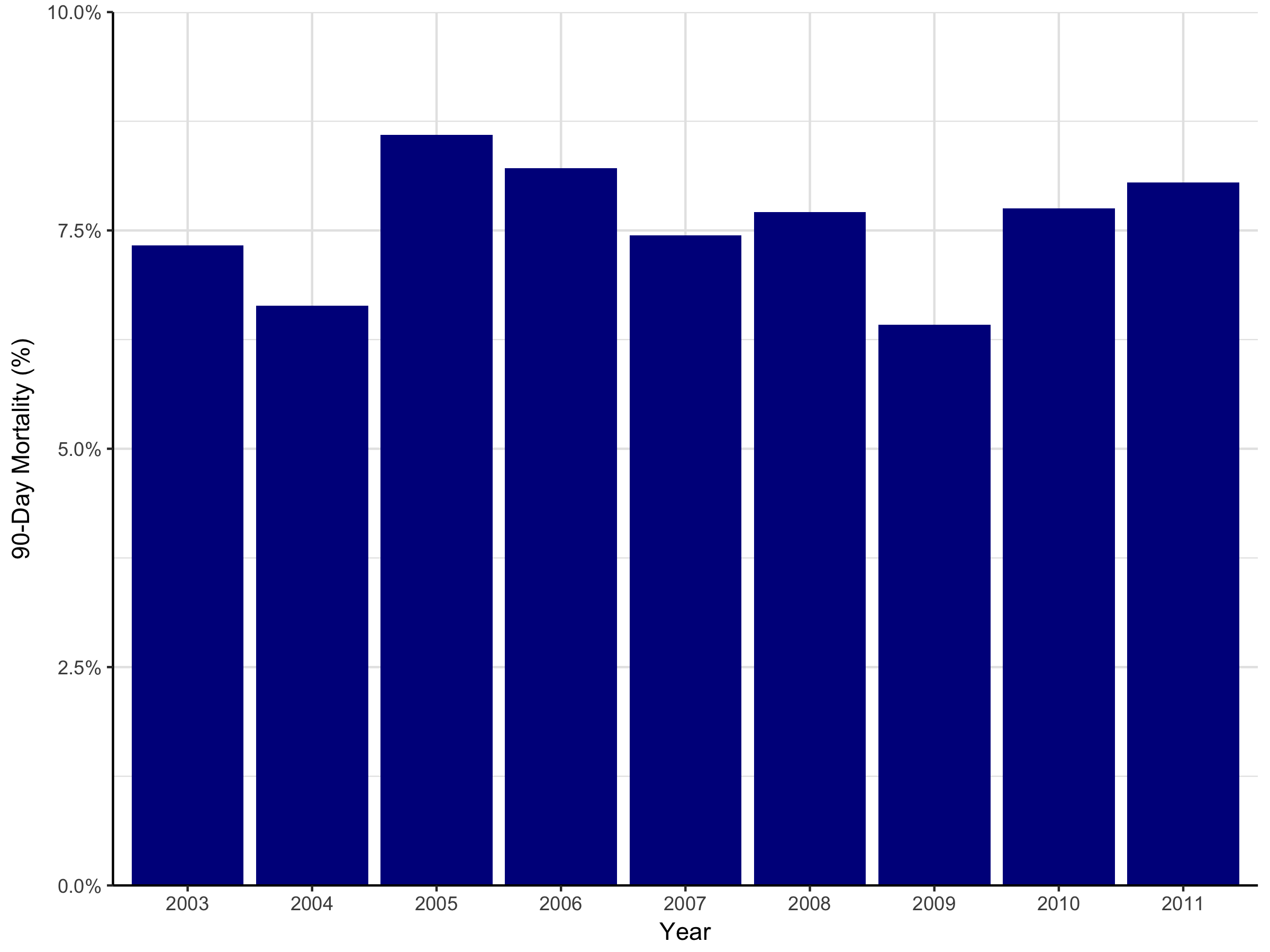


Supplemental Figure 3: Distribution of predicted 90-day mortality risk (%) stratified by annual cystectomy volume. Line at 10% delineates the upper quartile of predicted risk in all patients.


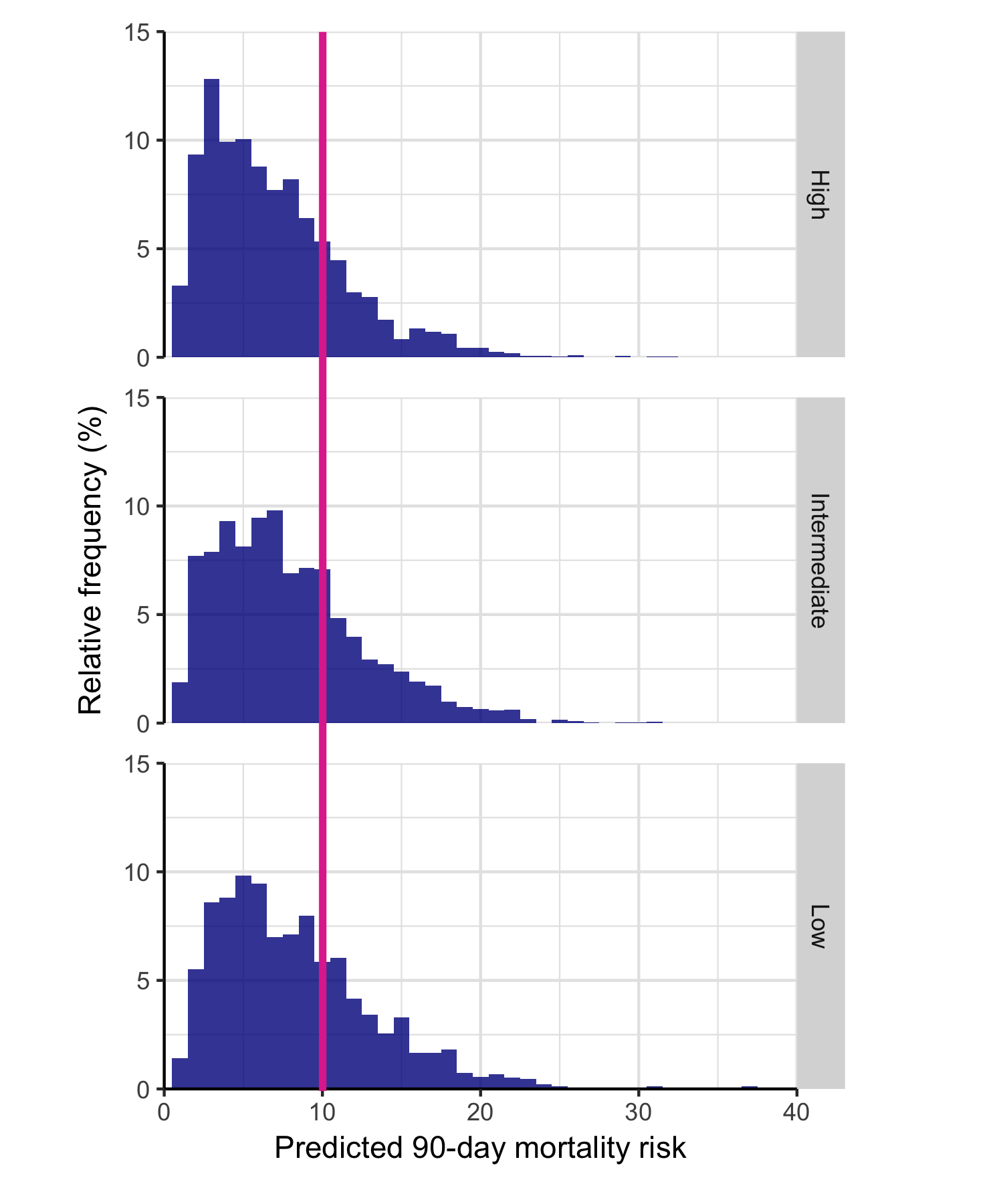

Supplement: Supplementary Data [file pky075_supp.docx]
